# Understanding the Electronic Transport of Al–Si and Al–Ge Nanojunctions by Exploiting Temperature-Dependent Bias Spectroscopy

**DOI:** 10.1021/acsami.3c18674

**Published:** 2024-04-02

**Authors:** Raphael Behrle, Corban G. E. Murphey, James F. Cahoon, Sven Barth, Martien I. den Hertog, Walter M. Weber, Masiar Sistani

**Affiliations:** † Institute of Solid State Electronics, Technische Universität Wien, Vienna 1040, Austria; ‡ Department of Chemistry, University of North Carolina at Chapel Hill, Chapel Hill, North Carolina 27599, United States; § Physics Institute, Goethe Universität Frankfurt, Frankfurt am Main 60438, Germany; ∥ Institute of Inorganic and Analytical Chemistry, Goethe Universität Frankfurt, Frankfurt am Main 60438, Germany; ⊥ Institut Néel, CNRS UPR2940, Grenoble 38042, France

**Keywords:** silicon, germanium, aluminum, metal-semiconductor heterostructure, Schottky barrier field-effect transistor

## Abstract

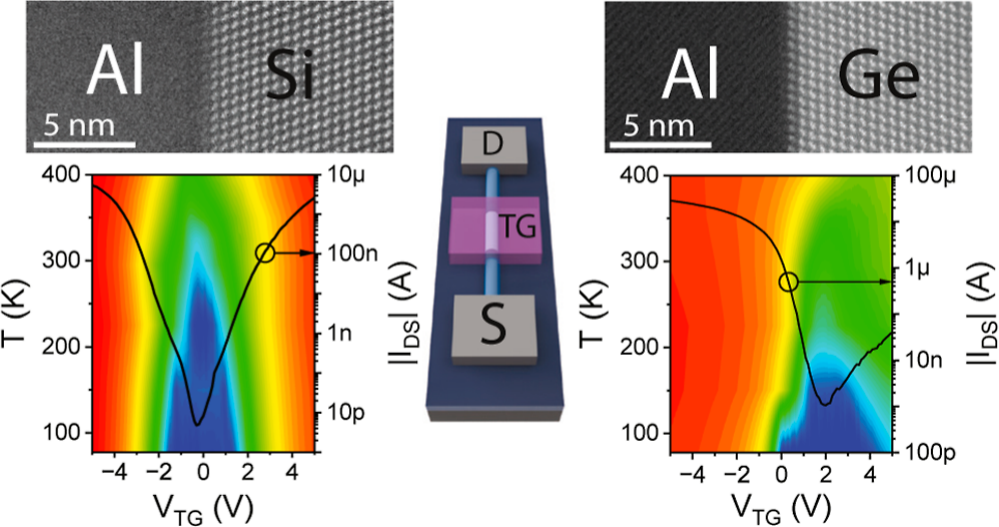

Understanding the electronic transport of metal–semiconductor heterojunctions is of utmost importance for a wide range of emerging nanoelectronic devices like adaptive transistors, biosensors, and quantum devices. Here, we provide a comparison and in-depth discussion of the investigated Schottky heterojunction devices based on Si and Ge nanowires contacted with pure single-crystal Al. Key for the fabrication of these devices is the selective solid-state metal–semiconductor exchange of Si and Ge nanowires into Al, delivering void-free, single-crystal Al contacts with flat Schottky junctions, distinct from the bulk counterparts. Thereof, a systematic comparison of the temperature-dependent charge carrier injection and transport in Si and Ge by means of current-bias spectroscopy is visualized by 2D colormaps. Thus, it reveals important insights into the operation mechanisms and regimes that cannot be exploited by conventional single-sweep output and transfer characteristics. Importantly, it was found that the Al–Si system shows symmetric effective Schottky barrier (SB) heights for holes and electrons, whereas the Al–Ge system reveals a highly transparent contact for holes due to Fermi level pinning close to the valence band with charge carrier injection saturation due to a thinned effective SB. Moreover, thermionic field emission limits the overall electron conduction, indicating a distinct SB for electrons.

## Introduction

Nanowire (NW)-based Schottky barrier (SB) metal–semiconductor–metal (MSM) heterostructures are highly interesting for emerging applications in nanoelectronics^1,2^ and quantum electronics^3,4^ that take advantage of their unique physical, electrical, and photonic as well as plasmonic properties.^5,6^ Having such a MSM structure allows to electrostatically tune the metal–semiconductor junctions as well as the channel’s energy landscape through its implementation in a SB field-effect transistor (SBFET).^2^ In this respect, undoped SBFETs show a certain degree of ambipolar charge carrier injection of electrons and holes into the channel, enabling dedicated “More than Moore” paradigms, e.g., reconfigurable FETs (RFETs).^7,8^ Importantly, the source/drain contact metal is of high relevance in defining the charge carrier injection capabilities. Here, the Fermi level pinning of the proposed material system is crucial, as it directly affects the SB exhibited to electrons and holes.^9,10^ In this respect, works considering Al as source/drain contacts demonstrated promising device concepts, reaching from Si RFETs^11^ to negative differential resistance devices based on Ge.^12^ From a material point of view, these Al contacts do not form intermetallic phases and provide abrupt and flat metal–semiconductor junctions to both Si and Ge, without the known deficiencies of bulk and layered Al–Si/Ge systems in the nanoscale.^13−15^ Importantly, SBFETs show unique characteristics that are fundamentally different from metal–oxide–semiconductor FETs (MOSFETs), allowing for systematic investigations of the injection of charge carriers into the semiconductor, i.e., transport over and/or tunneling through the SB, as well as the transport mechanisms of electrons and holes in the channel.^16,17^ In this respect, theoretical investigations on the charge carrier transport in Ni-silicide SBFETs were conducted.^18,19^ Utilizing the electrostatic tunability of the junctions and channel, the SB shape and its transmissivity can be modulated by varying bias conditions, allowing for the analysis of the transport characteristics of the metal–semiconductor heterostructure in more depth. Investigating the output characteristics over temperature of the NW SBFETs also allows to estimate the activation energy, i.e., the thermal injection capability of charge carriers into the semiconductor.^13,20^ Certainly, the theoretical first principles of TCAD modeling of the metal–semiconductor interface would even allow a more detailed analysis of the charge carrier injection contributions, such as thermionic emission (TE), thermally assisted field emission (TFE), and field emission (FE),^21^ which in an experimental approach can be merely didactically interpreted.

Importantly, the bias and the gate voltages as well as the temperature highly influence the electronic transport in different manners, which are difficult to analyze by single parameter sweeps. In this sense, the shape of the SB, the tunneling probability, as well as the injection mechanism itself varies and determines the transport. On this matter, bias spectroscopy is a powerful methodology to visualize a detailed image of the electronic transport and indicate different operation regimes due to the fact that multiple transport mechanisms are involved in the operation of SBFETs. Thus, temperature-dependent output and transfer I/V-characteristics are systematically visualized and evaluated. Using 2D colormaps for illustration allows one to obtain a comprehensive picture of the influence of the different parameters on the transport mechanisms. A key technological breakthrough that is allowing such measurements on Ge bottom-up NWs is a novel growth mechanism incorporating an intrinsic phenyl ligand surface passivation during synthesis (cf. Methods section).^22^ Till now such an in-depth electronic transport investigation of Al-group IV metal–semiconductor heterostructures is missing. Having physically and electrically equal Al leads to both Si and Ge provides a unique opportunity for insights and comparisons that might be useful for future applications of group IV SBFETs with monolithic Al contacts.

## Results and Discussion

**Figure 1:**
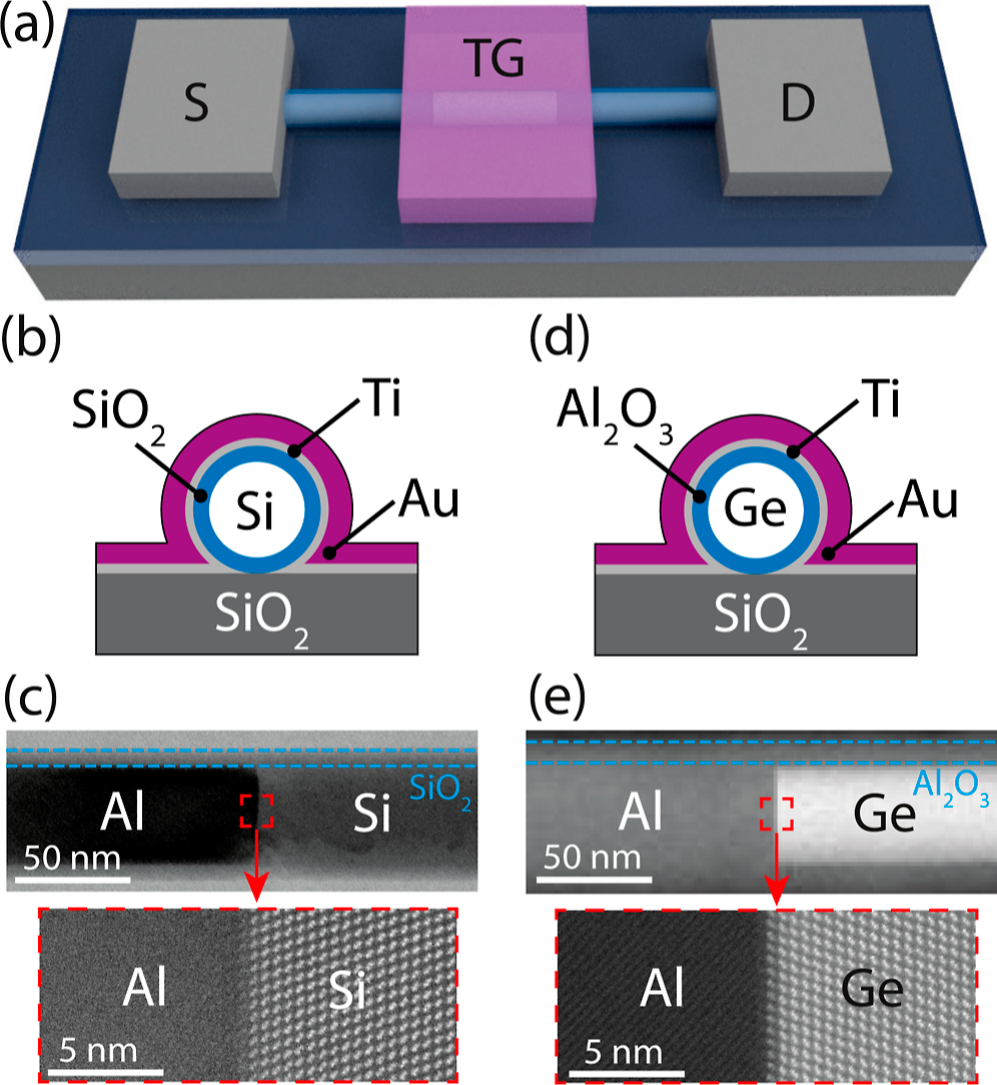
(a) 3D illustration schematically showing the SBFET architecture. The gate-stack for the Al–Si and Al–Ge devices is depicted in (b,d), respectively. Contrast-enhanced HAADF STEM images showing the junction abruptness of the Al–Si (c) and Al–Ge (e) junctions.

In this study, nominally intrinsic, bottom-up grown, Si and Ge NWs are contacted by single-crystalline Al leads, where a rapid thermal annealing (RTA) process initiates a metal–semiconductor exchange reaction that forms the desired MSM heterostructures (cf. Methods section).^13,23^ Note that prior to integration, the ⟨112⟩ Si NWs are passivated with a 9 nm thick thermally grown SiO_2_. Due to the absence of a stable native oxide, the ⟨111⟩ Ge NWs were conformly covered in a 10 nm thick atomic layer-deposited Al_2_O_3_ shell. Importantly, these passivation layers also act as gate oxide. Notably, Ge NWs as well as any Ge surface are prone to exhibit strong transient effects due to the presence of Ge_*x*_O_*y*_ at the semiconductor-high-κ (here: Al_2_O_3_) interface.^24,25^ In this respect, Hanrath and Korgel have identified “slow” interface trap states to be associated to an enhancement of the p-type behavior of Ge, whereas “fast” interface states are responsible for recombination processes.^26^ After drain/source contact formation (here: Al), a top-gate (TG), consisting of Ti/Au, covering the interfaces and the semiconducting channel is fabricated to realize a SBFET.^1,27^ Both the Al-Si-Al and Al-Ge-Al MSM structures exhibited a nominal *d*_NW_ of ≈70 nm and a *L*_NW_ of ≈1 μm (cf. Methods section). The 3D illustration in Figure 1a shows the fabricated devices. Additionally, the discussed gate-stack of the Al-Si-Al device is shown in Figure 1b and that of the Al-Ge-Al device in Figure 1d. Importantly, the same materials—except for the semiconductor NW and gate oxide—were used, allowing an appropriate comparison of the Al-group IV heterostructures. Applying a bias voltage to the drain/source contacts as well as the TG allows for the operation of the device in different regimes, enabling detailed investigations of the transport mechanism of the proposed material systems.

Bright field (BF) and high-angle annular dark-field (HAADF) scanning transmission electron microscopy (STEM) images of the Al–Si NW interface (cf. Figure 1c) reveal the abrupt and monolithic nature of the junction formed after the thermally induced exchange reaction, performed at a temperature of *T* = 773.15 K. In equal measure, but at a temperature of 673.15 K, the Al–Ge exchange mechanism is initiated (cf. Figure 1e). Note that detailed investigations on the Al–Si as well as on the Al–Ge exchange mechanism were carried out in the past.^13,23^

**Figure 2:**
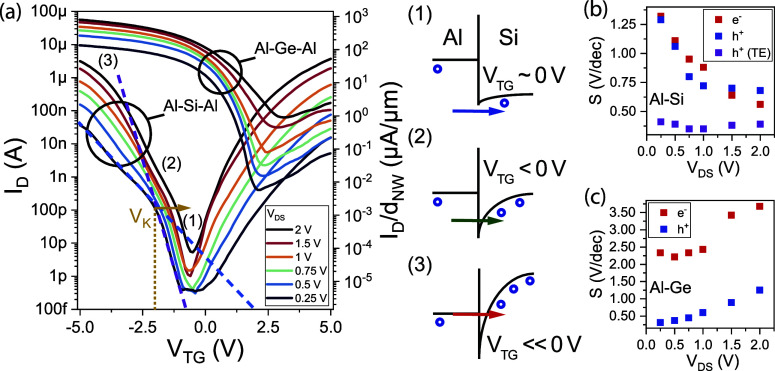
(a) Transfer characteristics at different *V*_DS_ at *T* = 295 K of the Al–Si and Al–Ge SBFETs. Notably, for *V*_DS_ = 0.25 V at *V*_TG_ = – 1.95 V of the Al–Si SBFET, a kink is evident, indicating the transition from TE to TFE/FE. Left to (a), (1–3) illustrate the different injection mechanisms (here: holes) in terms of simplified band diagrams. (b) Extracted steepest inverse subthreshold slopes S [Δ*V*_TG_/Δlog(*I*_D_)] of the Al–Si SBFET. The violet symbols show the slopes in the TE regime. (c) Slopes S of the Al–Ge SBFET increasing with higher applied bias *V*_DS_ due to the reduced bandgap of Ge.

Figure 2a shows the obtained transfer characteristics of the proposed Al–Si/Ge–Al SBFETs, sweeping the voltage *V*_TG_ from 5 to −5 V and applying drain/source biases *V*_DS_ ranging from 0.25 to 2 V. Note that *V*_D_ = −*V*_S_ and *V*_DS_ = *V*_D_ – *V*_S_. Analyzing the general appearance of the two sets of curves (Al–Si vs Al–Ge) reveals one of the main differences between the two material systems integrated in the proposed SBFET architecture. In the Al-Si-Al system, a relatively equal electron (*I*_D_ at *V*_TG_ = 5 V) and hole (*I*_D_ at *V*_TG_ = – 5 V) current is evident, whereas the hole transport (*V*_TG_ < 0 V) in the Al-Ge-Al system is dominant in comparison to the electron current. This can be attributed to the difference in the Fermi level pinning of the two systems, where the Fermi level at the Al–Si interface pins near the middle of the Si bandgap, leading to a dedicated electron and hole SB.^9^ In contrast, the Al–Ge interface has the Fermi level pinning close to the valence band, leading to dominant hole injection and thus to a highly transparent quasi-ohmic contact.^28,29^ In the transfer characteristic (cf. Figure 2a), the high transparency of the Al–Ge junction^30^ gets evident due to the relatively high and temperature-stable on-current at *V*_TG_ < 0 V. For further clarification, the output characteristic at *V*_TG_ < 0 V is investigated, revealing a highly linear behavior at low *V*_DS_ (cf. inset in Figure 5b). Additionally, the significantly lower bandgap of Ge [*E*_g_(Ge) = 0.67 eV] in comparison to Si [*E*_g_(Si) = 1.12 eV] becomes apparent due to the fact that the on- and off-currents also strongly depend on the SB height, which needs to be overcome by charge carriers. Therefore, the p-type currents of the Al–Ge material system are higher than those for the Al–Si material system.^31^ In this regard, electron and hole mobility would need to be considered, as high mobilities lead to higher drain currents and switching speeds. However, as extracting exact mobility values is difficult for bottom-up grown NWs^32^ due to their low gate oxide capacitance hindering C/V measurements,^33^ no values can be given for the NWs in this work. Another important aspect becomes evident by observing the steepness of the slopes [cf. Figure 2b (Si) and Figure 2c (Ge)]. Notably, in the p-branch of the Al-Si-Al device, a characteristic kink at the “kink” voltage *V*_K_ is observable, see Figure 2a, indicating the transition from TE (charge carrier injection over the barrier) to TFE/FE (charge carrier injection via tunneling) dominated currents,^16^ where the violet dashed line indicates the TE slope and the blue dashed line indicates the TFE/FE slope. As shown in Figure 2a, the different regimes can be associated with specific band bending grades and, thereof, injection mechanisms. Illustration (1) shows the case at *V*_TG_ ≈ 0 V, where TE is the dominant transport mechanism, as charge carriers can only overcome the SB for channel injection because band bending and therefore sufficient SB thinning are not present. Thus, tunneling does not contribute to charge carrier injection. As shown in (2), further bending the bands by operating the device in stronger accumulation (*V*_TG_ ≪ 0 V) TFE/FE gets more dominant due to the fact that the SB is getting thinner, allowing a stronger tunneling contribution, which gets evident by a shallower slope.^31^ Interestingly, *V*_K_ shifts from −1.95 V at *V*_DS_ = 0.25 V to *V*_K_ = – 1.16 V at *V*_DS_ = 2 V as the bands bend progressively downward by increasing *V*_DS_, and thus TFE/FE dominates the charge carrier injection at lower *V*_TG_ again due to stronger band bending. This scenario is illustrated in (3). In this context, it needs to be considered, that an increased number of charge carriers in the channel [cf. hole injection in (1–3), Figure 2a] further enhances the band bending, favoring the injection of charge carriers.^27^ Importantly, the transition from TE to TFE/FE was already described by simulations.^31,34^ In terms of theoretical investigations of the band bending mechanism, the charge carrier injection related to SBFETs as well as the electrostatic gating capabilities were also analyzed in depth by physics-based simulations by Roemer et al.^18,19^ Due to the use of transiently stable Si NWs and an expected low interface trap state density between Si and SiO_2_ as gate oxide, it is possible to show the transition from TE to TFE/FE with respect to a slope change here also experimentally. Figure 2b illustrates the steepest extracted inverse subthreshold slopes, S = Δ*V*_TG_/Δlog(*I*_D_) in the subthreshold regime of the Al–Si SBFET for electron and hole conduction. Importantly, in the TE transport, the SB height dominates the injection. Thus, the inverse subthreshold slope remains constant (violet symbols) in the range of ≈0.4 V/dec and shows that the device geometry does not suffer from short channel effects, such as drain-induced barrier lowering. In contrast, when TFE/FE is getting dominant, the slope becomes more shallow in comparison to TE due to tunneling transport.^20^ However, with higher applied drain/source bias (blue symbols: p-type and red symbols: n-type), the inverse subthreshold slope approaches values ranging from ≈1.3 V/dec at *V*_DS_ = 0.25 V to ≈0.6 V/dec at *V*_DS_ = 2 V. Due to stronger band bending induced by higher applied biases, the tunneling barrier becomes thinner and thus enables more efficient injection of charge carriers; see (3). Remarkably, this kink is merely visible in the p-branch of the transfer characteristic and is not observable in the n-branch (*V*_TG_ > 0 V). We speculate that a slightly higher SB height evident for electrons leads to diminishing of the kink in the n-branch. In contrast, the Al–Ge SBFET does not exhibit a kink in the slope due to the quasi-ohmic contact for the injection of holes (cf. Figure 2c). Nevertheless, the kink could also be present, but could fall into the increased source/drain leakage and thus not be visible in the I/V data. Remarkably, here the slope increases, applying higher *V*_DS_ as the smaller bandgap leads to a significant increase in the off-current caused by an increased number of charge carriers in the channel, screening the transition from TE to TFE/FE. In contrast to the relatively symmetric slopes of the n- and p-branch of the Al–Si SBFET devices, the slopes for the Al–Ge SBFET differ, which is an indication of different injection capabilities of electrons and holes, mainly caused by the differences of the Fermi level pinning of the systems. Considering the Al–Si material system, a close-to-midgap pinning is evident, causing similar SBs for electrons and holes, whereas for Al–Ge junctions, the Fermi level pins are close to the valence band of Ge.^9^ Therefore, in the Al–Ge material system, a dominant p-type behavior is observed, also causing differences in the inverse subthreshold slope characteristics in comparison to the Al–Si material system.^31^

**Figure 3:**
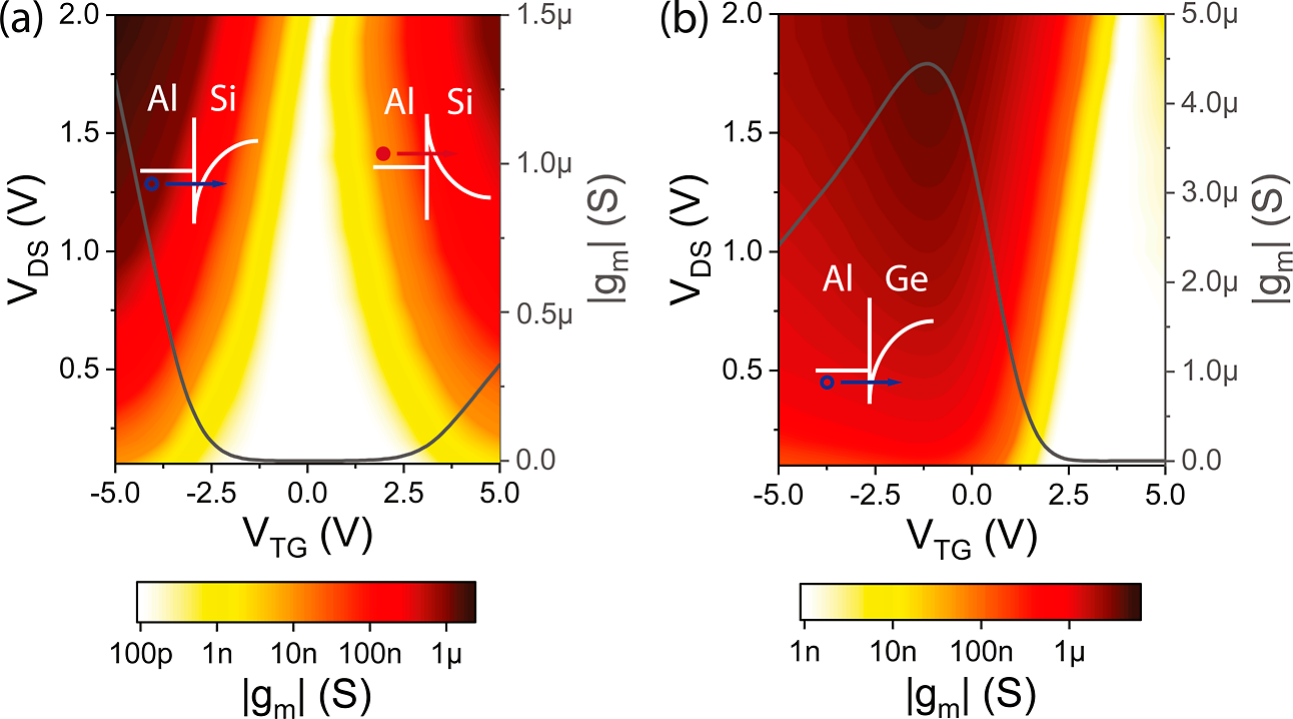
Transconductance |*g*_m_| maps with *V*_DS_ ranging from 0.1 to 2 V derived from the transfer characteristics of an (a) Al–Si SBFET and an (b) Al–Ge SBFET. Additionally, *g*_m_ vs *V*_TG_ at *V*_DS_ = 1 V is plotted as a solid line and indicated on the right gray *y*-axis in both plots. The insets depict schematic band diagrams of both systems.

Furthermore, analyzing the transfer characteristics (cf. Figure 2a) allows us to extract the transconductance *g*_m_ = d*I*_D_/d*V*_TG_. Consequently, we give a qualitative measure of the input to output response in terms of the inverse subthreshold slope and thus an in-depth insight on the transport phenomena of the proposed material systems. Figure 3 shows the transconductance *g*_m_ extracted with *V*_DS_ ranging from 0.1 to 2 V and *V*_TG_ ranging from −5 to 5 V for the Al–Si (Figure 3a) and the Al–Ge (Figure 3b) SBFETs. Interestingly, analyzing the Al–Si SBFET, an asymmetric |*g*_m_| value at |*V*_TG_| = 5 V is obvious which correlates with the transfer characteristic shown in Figure 2a. At *V*_TG_ ≫ 0 V, |*g*_m_| values of 313 nS (4.35 μS/μm) and 1.27 μS (17.64 μS/μm) are achieved in the n- and p-type branch, respectively, at *V*_DS_ = 1 V. Another important feature of the Al–Si SBFET is the steady slope of *g*_m_, even at |*V*_TG_| = 5 V, required for electronic devices, e.g., amplifiers.^35^ Here, we assume *g*_m_ saturation for higher |*V*_TG_|. In contrast to the Al–Si SBFET, the transconductance *g*_m_ of the Al–Ge SBFET shown in Figure 3b shows no steadily increasing slopes in strong accumulation, instead exhibiting a degradation of *g*_m_ in the p-branch. Interestingly, such a degradation is typically visible in SBFETs with distinct barriers at specific bias conditions.^17^ In this context, it needs to be distinguished between junction and channel resistance contributions, whereas *g*_m_ degradation is commonly attributed to surface roughness scattering as well as to the total resistance (junction resistance and channel resistance). In the Al–Ge SBFET, the Fermi level pins are close to the valence band (see inset in Figure 3b) and thus enable efficient injection of holes into the semiconductor. In this case, the saturation and consequently the degradation becomes evident at any bias condition independently of the applied *V*_DS_. Therefore, it can be assumed that the highly transparent (quasi-ohmic) junction with phonon scattering in the Ge channel is the predominant factor in this context.^36^ Moreover, higher *g*_m_ values are reached in the p-branch (*V*_TG_ = – 5 V) of the Al–Ge SBFET with 2.44 μS (32.53 μS/μm) in comparison to the Al–Si SBFET (1.27 μS) at *V*_DS_ = 2 V, which can be attributed to steeper slopes of the p-branch transfer curves (cf. Figure 2c) in the Al–Ge SBFET within the observed regime. Note that the degradation in strong accumulation leads to a maximum *g*_m_ = 4.43 μS (59.07 μS/μm) at *V*_TG_ = – 1.19 V. Remarkably, the *g*_m_ maximum even occurs at low |*V*_TG_| values, caused by a shift of the transfer characteristic (cf. Figure 2a) evident in the Al–Ge system, which can be elaborated by “slow” surface trap states induced shifting toward more dominant p-type behavior.^24^ Due to weak electron conduction, in consequence of a relatively high barrier for electrons, *g*_m_ merely reaches a value of 2.6 nS (0.03 μS/μm) at *V*_TG_ = 5 V.

**Figure 4:**
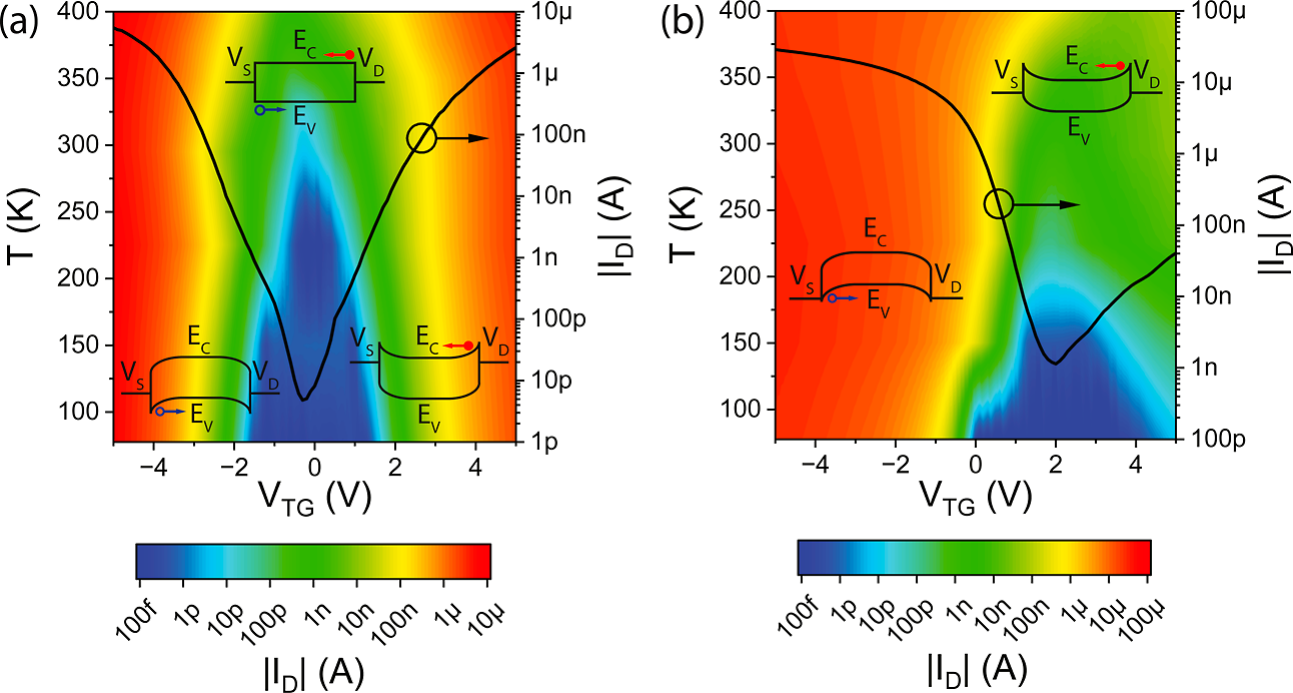
Temperature-dependent transfer characteristic maps of an (a) Al–Si and an (b) Al–Ge SBFET at *V*_DS_ = 1 V. The black curve belonging to the right *y*-axis shows the transfer characteristic at *T* = 295 K. The insets illustrate the schematic band diagrams of the corresponding predominant transport regimes.

Next, Figure 4 shows the temperature-dependent transfer characteristics of both the Al–Si and Al–Ge SBFETs at *V*_DS_ = 1 V. Indeed, due to the relatively symmetric Fermi level pinning of Al to Si, a symmetric transport, with *V*_TG_ = 0 V being the vertical symmetry axis, is visible, which is confirming our initial assumption. Note that the insets shown in Figure 4a illustrate the schematic band diagrams in the corresponding *V*_TG_ regimes without an applied source/drain bias. Importantly, at *V*_TG_ = 0 V, dedicated barriers for electrons as well as holes are evident, leading to low off-currents in the 100 fA-regime (minimum resolution of the measurement equipment). Elevating the temperature leads to an increased off-current due to thermally excited charge carriers. However, considering the current in the n- and p-branch, transiently stable operation modes are observed over the investigated temperature regime from 77.5 to 400 K, proving sufficient operation of the Al–Si material system in the investigated regime. The Al–Ge system, shown in Figure 4b, reveals strong temperature dependencies, which can be attributed to the reduced bandgap of Ge in comparison to Si as well as strong Fermi level pinning close to the valence band, leading to a high contribution of thermally excited charge carriers easily overcoming the low SB exhibited to holes. Moreover, the facts of a smaller bandgap as well as lower SB are becoming apparent, observing the off-current, which shows a stronger temperature dependency in comparison to the Al–Si material system and distributes over a wider *V*_TG_. Considering the current in the p-branch, remarkably high values in the 10 μA-regime are evident, further revealing dominant p-type characteristic of the Al–Ge SBFET.

**Figure 5:**
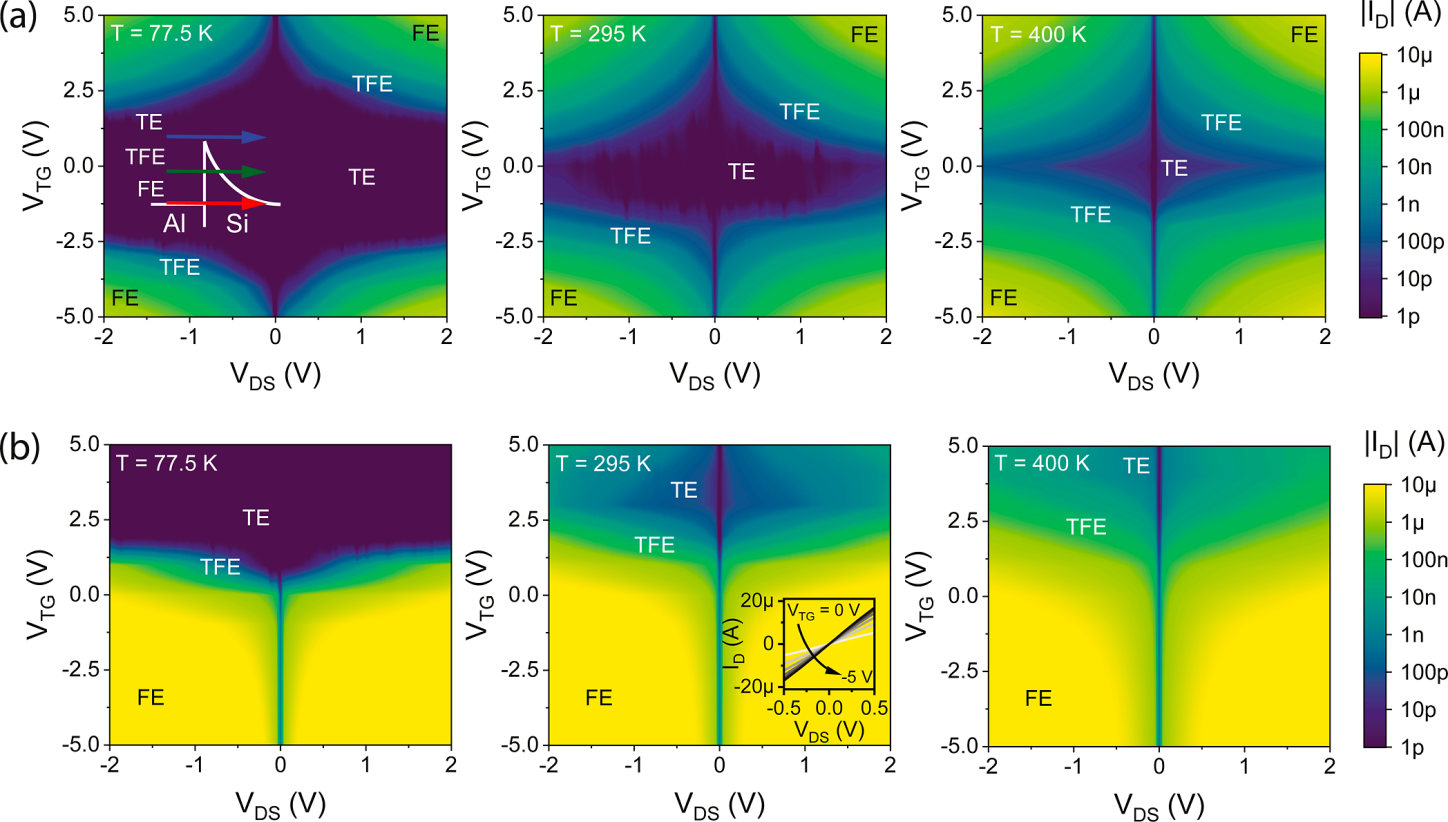
Output characteristic maps in dependence of the applied TG voltage *V*_TG_ at *T* = 77.5, 295, and 400 K for an (a) Al–Si SBFET and an (b) Al–Ge SBFET, respectively. TE, TFE, and FE regimes are didactically stated at the corresponding regimes. The inset in (a) illustrates the injection mechanism for electrons. The inset in (b) at *T* = 295 K illustrates the linear behavior of the output characteristic at low *V*_DS_ with *V*_TG_ ranging from 0 to −5 V, indicating quasi-ohmic Al–Ge junctions.

**Figure 6:**
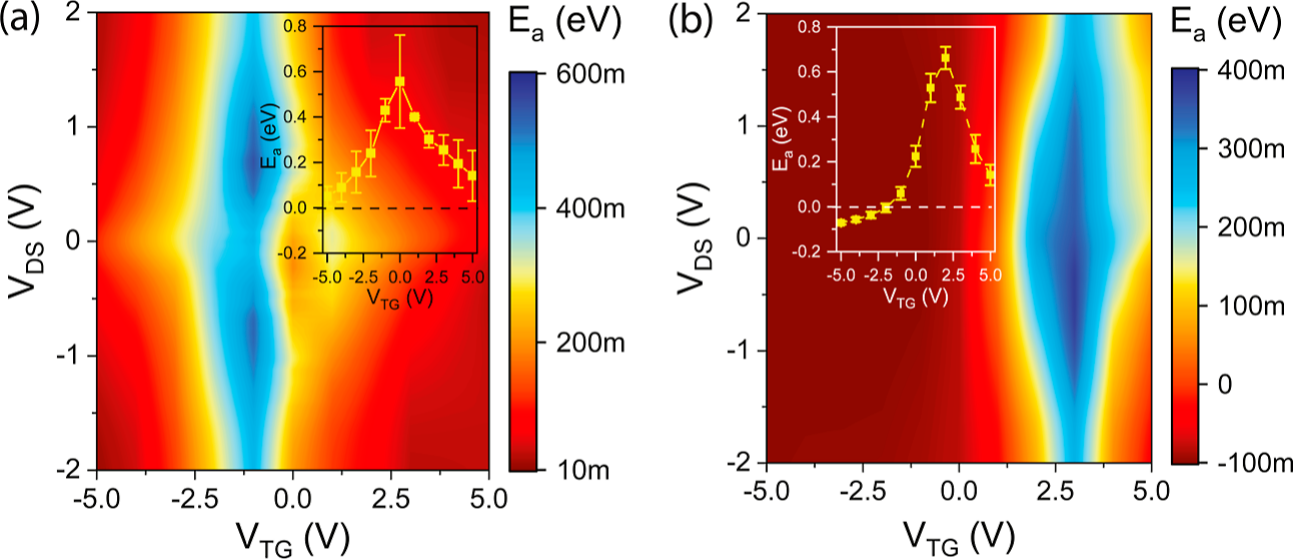
Activation energy maps extracted from output I/V data over temperature of the (a) Al–Si and the (b) Al–Ge SBFET. The insets show the intrinsic activation energy in dependence of *V*_TG_. The error bars represent the standard deviation of three similar SBFETs for both material systems.

For more detailed investigations on the bias-dependent transport, the output characteristics at different temperatures of the Al–Si (Figure 5a) and Al–Ge (Figure 5b) SBFETs are analyzed. The inset in Figure 5a (Al–Si at *T* = 77.5 K) illustrates the three main contributions relevant to charge carrier injection. Here, TE is the contribution originating from charge carriers injected over the SB barrier into the semiconductor; see also band diagram (1) in Figure 2a. Importantly, this contribution increases with elevated temperatures due to thermally excited charge carriers overcoming the barrier. Next, FE is considered, which is the contribution originating from the Fermi level pinning of the metal into the semiconductor through tunneling. Here, the charge carriers exhibit minimal potential energy but are efficiently injected in the case of high biases (here: |*V*_DS_| = 2 V and |*V*_TG_| = 5 V) consequently leading to strong band bending and thus thinner tunneling barriers enabling efficient injection of charge carriers, see also band diagram (3) in Figure 2a. In this context, the transmissivity *T*_FE_ in the case of FE—and considering WKB approximation—is given as , where m* is the tunneling effective mass, Φ is the SB height, and ε is the electric field, which directly influences the barrier width. Finally, TFE is a mixed transport mechanism of TE and FE, where thermally excited charge carriers can tunnel through the remaining SB; see also band diagrams (2) and (3) in Figure 2a. Again, applying higher biases, i.e., thinning the tunneling barrier, allows an efficient injection via this transport mechanism. The output characteristics of the Al–Si SBFET (cf. Figure 5a) reveal a vertical (*V*_DS_ = 0 V) and a horizontal (*V*_TG_ = 0 V) symmetry axis, further proving the symmetrical nature of the Al–Si system. At T = 77.5 K, charge carrier transport is merely possible via TFE and FE due to frozen-out charge carriers not being able to overcome the barrier caused by the lack of thermal excitation. However, elevating the temperature, thus thermally exciting charge carriers, leads to carrier injection even at lower bias voltages. In comparison, the Al–Ge SBFET exhibits only one vertical symmetry axis at *V*_DS_ = 0 V due to its asymmetric Fermi level pinning, with the Fermi level being close to the valence band. Considering *T* = 77.5 K, only a negligible increase in the current with increasing |*V*_DS_| is observable, which is related to minimal thermal excitation and the dominance of the quasi-ohmic contact between Al and Ge, which is defined by dominant thermal-independent tunneling.^14^ Given the combination of a low SB height and efficient band bending yielding an ultrathin barrier, most holes seem to tunnel into the valence band with a high transmission probability. Evidence of this is given in Figure 6b, where near-zero and slightly negative effective SB heights were extracted.^30^ We interpret this as a strong indicator for quasi-ohmic contacts, as charge carriers (here: holes) do not experience a thermal energy barrier to overcome the metal–semiconductor junction. Furthermore, an increase in *V*_TG_ and/or *V*_DS_ does not lead to sufficient band bending, enabling TFE/FE in this regime. Analyzing the transport at elevated temperatures shows that the FE remains temperature-independent, but the TE in the n-branch (*V*_TG_ ≫ 0 V) increases due to thermally excited charge carriers.

Finally, upon processing the output I/V data shown in Figure 5, the activation energy *E*_a_ of the material systems can be evaluated (see Figure 6). The basis for the calculation is the TE current model adapted to be suitable for the experimental approach used here. Hence, the voltage between the metal and semiconductor cannot be considered due to the experimental setup, and a simplified model needs to be applied, which is valid for an applied bias voltage exceeding 3*k*_B_*T*/*q* (76 mV at *T* = 300 K).^37^ Consequently, , where *J*_TE_ is the measured current density, A* is the effective Richardson constant, *T* is the corresponding temperature, and *E*_a_ is the total effective activation energy.^37^ Note that this model does not allow a distinction between the proposed transport mechanisms, as in the experimental setup, the total current is measured and thus merely gives an estimation of *E*_a_. Rearranging the equation accordingly, a Richardson plot is obtained allowing the extraction of the activation energy, i.e., the energy required to inject charge carriers into the semiconductor. Using this approach, the *V*_TG_-dependent intrinsic activation energy is obtained (cf. insets in Figure 6) by fitting *E*_a_ to *V*_DS_ = 0 V. Moreover, performing a separate measurement with a wider *V*_DS_ range and without fitting to *V*_DS_ = 0 V, a 2D colormap in dependence of *V*_TG_ and *V*_DS_ can be created, as shown in Figure 6. As shown in the map and in the inset, the activation energy *E*_a_ of the Al–Si SBFET (cf. Figure 6a) exhibits positive and similar *E*_a_ values at any measured operation point, indicating dedicated SBs for electrons and holes, further demonstrating the relative midgap pinning of Al to the Si bandgap. Remarkably, considering the intrinsic *E*_a_ (cf. inset of Figure 6a), a vertical symmetry axis at *V*_TG_ = 0 V is visible. Notably, in the off-state of the Al–Si SBFET, a relatively high activation energy is evaluated, corresponding well with previously shown results (cf. Figure 2a). In comparison, the Al–Ge SBFET shown in Figure 6b exhibits negative *E*_a_ values for hole conduction, further supporting the presence of quasi-ohmic contacts in the p-branch, caused by strong Fermi level pinning close to the valence band.^28,38^ This was also shown in carbon nanotubes with Pd contacts also claiming quasi-ohmic injection of charge carriers.^39^ Observing electron conduction at positive *V*_TG_ reveals that a dedicated barrier becomes evident. Moreover, a shift of the off-state into the n-branch takes place, further enhancing the p-branch and thus favoring hole conduction, which is caused by “slow” interface traps.^24^ Additionally, the use of Al_2_O_3_ as gate oxide (cf. Figure 1d,e) is further pushing the Al–Ge SBFET to dominant p-mode operation.^40^

## Conclusions

In conclusion, we have analyzed Al–Si and Al–Ge-based SBFETs in terms of temperature-dependent bias spectroscopy and provided an in-depth analysis of the involved electronic transport mechanisms. Remarkably, using single-crystalline Al as drain/source contacts allows for a comparison of the transport properties of Si and Ge SBFETs. Output and transfer characteristic measurements and systematic evaluations allow us to investigate the effects of Fermi level pinning, which in the Al–Ge SBFET leads to dominant p-type conduction and quasi-ohmic contacts for hole conduction, while Si-based SBFETs show equal charge carrier injection capabilities of electrons and holes. In comparison to single parameter sweeps, evaluating *g*_m_-, *I*_D_-, and *E*_a_-maps allows us to gain important insights into the dependence of various parameters directly influencing the transport behavior. Most notably, the presented investigations of the bias and temperature-dependent transport in Al–Si and Al–Ge nanojunctions contribute to a better understanding of the operation regimes and transport mechanisms of metal-group IV-based SBFETs, which are highly anticipated for the implementation of electronic device functionalities beyond the capabilities of conventional FETs and CMOS devices in general.

## Methods

### Si NW Growth

The ⟨112⟩ oriented intrinsic Si NWs were grown in a home-built, hot-wall chemical vapor deposition (CVD) system described previously using silane and HCl gases, hydrogen carrier gas, and Au nanoparticle catalysts of diameter 80 nm. NW growth was performed at 753 K with 2 standard cubic centimeters per minute (sccm) of SiH_4_, 4 sccm of HCl, and 194 sccm of H_2_ at 20 Torr total reactor pressure for 20 min until the desired NW length of 20 μm was obtained. The Si NWs have diameters *d*_NW_ of ≈70 nm. Subsequent to the growth, the Si NWs were thermally oxidized at *T* = 1174 K in O_2_ atmosphere for 3 min and annealed for another 3 min in N_2_ atmosphere to form a high-quality 9 nm thick SiO_2_ gate oxide.

### Ge NW Growth

The intrinsic Ge NWs were grown on Ge(111) single-crystal substrates by low-pressure CVD in a cold-wall reactor. Prior to use, the substrates were coated with a 1 nm thick Au film by sputtering. The Ge substrate was heated to 748 K under dynamic vacuum before diphenylgermane (DPG; 40 mg DPG reservoir at 295 K; process pressure <1 × 10^–3^ mbar) was introduced to the CVD chamber for 20 to 30 min, keeping the precursor at room temperature and applying dynamic vacuum. The Au seeds act as catalytic sites for the precursor decomposition. Similar growth procedures using the same precursor under LPCVD conditions have been reported in the literature.^22,41^ After the growth of the ≈70 nm thick Ge NWs, they were conformally coated with 10 nm of Al_2_O_3_ by atomic layer deposition at a temperature of T = 474 K.

### SBFET Fabrication

The passivated Si/Ge NWs were drop-casted onto a 100 nm thick thermally grown SiO_2_ layer atop a 500 μm thick highly p-doped Si substrate. Al contacts to the Si/Ge NWs were fabricated by a combination of electron beam lithography, 15 s of BHF (7:1) etching to remove the SiO_2_/Al_2_O_3_ shell at the contact area, 125 nm Al sputter deposition, and lift-off techniques. A successive thermally induced exchange reaction by RTA at a temperature of *T* = 774 K/*T* = 674 K in forming-gas atmosphere initiates the substitution of Si/Ge by Al. After annealing, the semiconductor channel length was ≈1 μm in length with a NW diameter of ≈70 nm for the Si NWs as well as Ge NWs (without passivation). Facilitating this heterostructure formation scheme allows the integration of single-crystalline monolithic Al–Si/Al–Ge NW heterostructures. The Ω-shaped Ti/Au TG covering the interfaces as well as semiconductor channel was fabricated using a combination of electron beam lithography, Ti/Au evaporation (10 nm Ti, 100 nm Au), and lift-off techniques.

### BF and HAADF STEM

BF and HAADF STEM (zoom) was performed on Al-Si-Al and Al-Ge-Al NW heterostructures fabricated on 40 nm thick Si_3_N_4_ membranes using a probe-corrected FEI Titan Themis, working at 200 kV. The Al–Si interface in the shown images is viewed along the [110] direction of observation of the Si crystal. {111} planes are visible in the Al region. HAADF images of the Al–Ge interface are shown, where the Ge crystal is in the [110] direction of observation. Similarly, {111} planes are visible in the Al region.

### Electrical Measurements

The electrical measurements were performed by using a combination of a semiconductor analyzer (HP 4156B) and a probe station. To minimize the influence of ambient light, as well as electromagnetic fields, the probe station is placed in a dark box. For applying the bias voltage, the following holds: *V*_DS_ = *V*_D_ – *V*_S_ and *V*_D_ = −*V*_S_. Temperature-dependent measurements as well as measurements to extract the activation energy were performed in a vacuum using a cryogenic probe station (Lake Shore PS-100) and a semiconductor analyzer (Keysight B1500A).
